# Effects of Cold-Rolling/Aging Treatments on the Shape Memory Properties of Ti_49.3_Ni_50.7_ Shape Memory Alloy

**DOI:** 10.3390/ma10070704

**Published:** 2017-06-26

**Authors:** Shih-Hang Chang, Keng-Hua Lin, Shyi-Kaan Wu

**Affiliations:** 1 Department of Chemical and Materials Engineering, National I-Lan University, I-Lan 260, Taiwan; shchang@niu.edu.tw; 2 Department of Materials Science and Engineering, National Taiwan University, Taipei 106, Taiwan; linkenghua@gmail.com

**Keywords:** shape memory alloy, thermomechanical treatment, theoretical calculation, texture, precipitation

## Abstract

In this study, the combined effects of strengthening, precipitates, and textures on the shape recovery ability and superelasticity of thermomechanically treated Ti_49.3_Ni_50.7_ shape memory alloy (SMA) in both the rolling and transverse directions were studied by experimental measurements and theoretical calculations. Experimental results and theoretical calculations showed that the 300 °C × 100 h aged specimen exhibited the best shape memory effect because it possessed the most favorable textures, highest matrix strength, and most beneficially coherent stress induced by Ti_3_Ni_4_ precipitates. The 30% cold-rolled and then 300 °C × 100 h aged specimen exhibited the highest strength and superelasticity; however, its shape recovery ability was not as good as expected because the less favorable textures and the high strength inhibited the movements of dislocations and martensite boundaries. Therefore, to achieve the most optimal shape memory characteristics of Ni-rich TiNi SMAs, the effects of textures, matrix strength, and internal defects, such as Ti_3_Ni_4_ precipitates and dislocations, should all be carefully considered and controlled during thermomechanical treatments.

## 1. Introduction

Near-equiatomic TiNi shape memory alloys (SMAs) are widely used in a variety of applications because of their excellent shape memory effect, superelasticity, high strength and ductility, and good damping capacity [1,2]. It has been reported that thermomechanical treatments, including work hardening, solid-solution strengthening, precipitation hardening, and grain refinement, normally strengthen TiNi SMAs by increasing the critical shear stress for slip [3,4,5]. In addition, the shape memory effect and superelasticity of thermomechanically treated TiNi SMAs can be improved by suppressing the irreversible slip deformation during the martensite reorientation and stress-induced martensitic transformation. Nevertheless, thermomechanical treatments may simultaneously change the textures and microstructures of TiNi SMAs, thereby influencing their mechanical properties. Numerous studies have reported the relationship between shape memory behaviors and the crystallographic properties of TiNi SMAs [6,7,8,9,10,11]. Miyazaki et al. [6] calculated the theoretical recoverable strains of single-crystal TiNi SMA along different directions by the lattice deformation matrix and compared these values to experimental results. Shu et al. [7] and Inoue et al. [8] calculated the theoretical recoverable strains of polycrystal TiNi SMAs possessing different textures and compared these values to experimental results. Ye et al. [9] observed the texture evolution of TiNi SMAs during thermal cycling under load and calculated the strain-texture map in B19’ martensite. Laplanche et al. [10] studied the evolutions of microstructure and texture during the processing of Ti_49_Ni_51_ shape memory sheets using electron backscatter diffraction. The effects of temperature and texture on the reorientation of martensite variants in TiNi SMAs have also been reported [11]. Even though these researchers thoroughly investigated the relationship between the texture and theoretical recoverable strain of SMAs, few researchers have considered the effects of microstructure, such as precipitates, dislocations, and the types of martensite twin variants, on the shape memory characteristics of SMAs. Therefore, these theoretical calculated values may deviate from the experimental results and lack practical application.

To address this issue, Sehitoglu et al. [12,13,14,15,16,17] conducted a series of investigations on the mechanical properties and theoretical recoverable strain of single crystal Ni-rich TiNi SMAs. They also studied the effects of precipitates on the shape memory properties of peak-aged and over-aged Ni-rich TiNi SMAs. Ni-rich TiNi SMAs were chosen for their studies because Ti_3_Ni_4_ normally precipitates when the alloys are aged at appropriate temperatures and time intervals. In the early stage of aging, the boundaries of the Ti_3_Ni_4_ precipitates are coherent with the matrix, and the lattices around these precipitates are distorted by the coherent stress field. The extent of the lattice distortion is larger in the longitudinal direction of the precipitates than in the transversal direction. As the aging time increases, Ti_3_Ni_4_ precipitates grow larger and lose the coherent interfacial relation with the matrix. Meanwhile, dislocations can be observed in the matrix around the precipitates. In addition, Ti_3_Ni_4_ precipitates can increase the matrix strength [18]. Nishida et al. [19] investigated the transformation behaviors of Ti_49_Ni_51_ SMA after aging and discovered that the morphologies of martensites in solution-treated and aged specimens are quite different. In solution-treated specimens, martensite variants are self-accommodated to each other. However, in aged specimens, the plate-like martensite grows along specific directions, and single-oriented martensite forms in the grains of the parent phase. The microstructures of most of the martensites in solution-treated specimens are <011>_M_ type II twins or ${\left(11\bar{1}\right)}_{M}$ type I twins, and those in aged specimens, which have fine lenticular Ti_3_Ni_4_ precipitates, are ${\left(001\right)}_{M}$ type I twins, with <011>_M_ type II twins appearing as the Ti_3_Ni_4_ precipitates grow larger.

However, because Sehitoglu’s research focused only on single crystals [12,13,14,15,16,17], no discussion was presented on the textures of the polycrystal effect. In practical applications, on the other hand, the transformation behaviors or mechanical properties of TiNi SMAs are often controlled by thermomechanical treatments. Although these treatments have several effects on TiNi SMAs and lead to complexity in comprehending the corresponding evolution of shape memory characteristics, these crucial effects have not yet been discussed systematically. In the present study, we aimed to understand the strengthening effects and the changes in microstructure and texture generated by thermomechanical treatments, and to elucidate their combined effects on the shape memory characteristics of Ti_49.3_Ni_50.7_ SMA. In addition, the theoretical recoverable strains of polycrystalline Ti_49.3_Ni_50.7_ SMA were calculated to elucidate the effects of textures on the shape memory characteristics of this alloy.

## 2. Results

### 2.1. DSC and Microhardness Results

**Figure 1:**
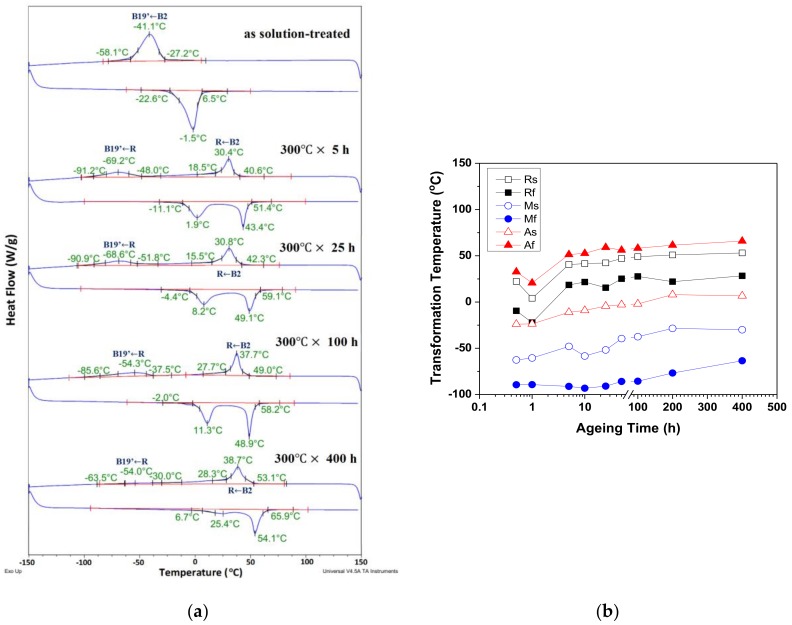
(**a**) DSC results of solution-treated and 300 °C aged Ti_49.3_Ni_50.7_ SMA for various time intervals from 0 to 400 h; (**b**) Variation of Ms, Mf, As, Af, Rs, and Rf transformation temperatures for Ti_49.3_Ni_50.7_ SMA with different aging times determined from Figure 1a. DSC: differential scanning calorimetry; SMA: shape memory alloy; Ms & Mf: the start and finish temperatures of forward martensitic transformation; As & Af: the start and finish temperatures of reverse martensitic transformation; Rs & Rf: the start and finish temperatures of R-phase transformation during the cooling.

Figure 1a shows the differential scanning calorimetry (DSC) results of the solution-treated and selected 300 °C aged Ti_49.3_Ni_50.7_ specimens for time intervals of 0 to 400 h. As shown in Figure 1a, the solution-treated specimen possessed a one-stage B2↔B19’ martensitic transformation, and all 300 °C aged specimens exhibited a two-stage B2↔R↔B19’ transformation. The presence of R-phase in aged Ti_49.3_Ni_50.7_ SMA was induced by the formation of Ti_3_Ni_4_ precipitates. Figure 1b shows the variation of Ms, Mf, As, Af, Rs, and Rf transformation temperatures (Ms and Mf are the start and finish temperatures of forward martensitic transformation, respectively; As and Af are those of reverse martensitic transformation, respectively; Rs and Rf represent the start and finish temperatures of R-phase transformation during the cooling, respectively) for 300 °C aged specimens determined from Figure 1a. As shown in Figure 1b, the transformation temperatures of all the 300 °C aged specimens increased with increasing aging time. 

**Figure 2:**
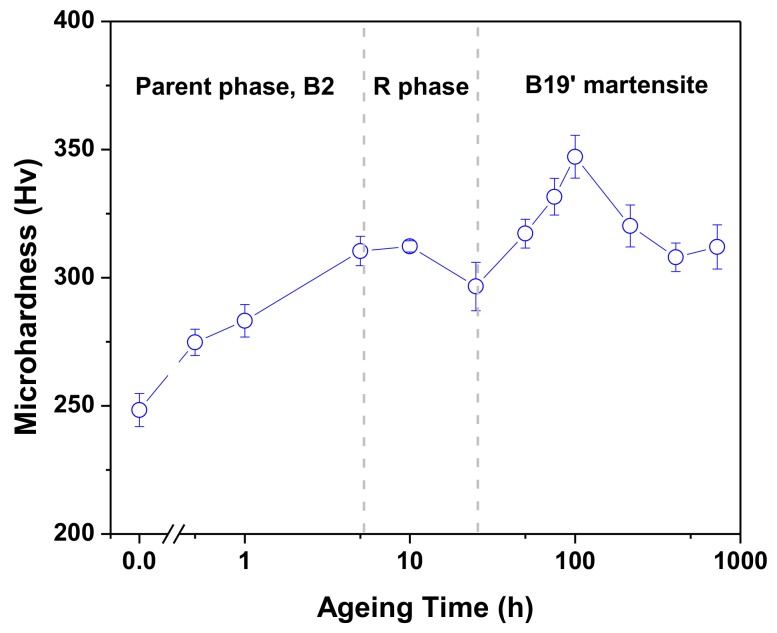
Micro Vickers hardness results of solution-treated and 300 °C aged Ti_49.3_Ni_50.7_ SMA.

Figure 2 plots the microhardness measurements of solution-treated and 300 °C aged Ti_49.3_Ni_50.7_ specimens. Figure 2 reveals that the microhardness of Ti_49.3_Ni_50.7_ SMA initially increased with increasing aging time due to the increasing amount of Ti_3_Ni_4_ precipitates in the aged specimens. However, the microhardness exhibited a drop during the aging time from 5 to 25 h because, in this aging period, the 300 °C aged specimens were in the soft R-phase state at room temperature, rather than in the hard B2 parent phase state, as demonstrated in the DSC results shown in Figure 1. After 25 h of aging, the microhardness of aged Ti_49.3_Ni_50.7_ specimens increased again with increasing aging time, approaching a maximum value of 347 Hv at 100 h. This suggests that aging for 100 h is the most beneficial aging treatment to improve the strength of Ti_49.3_Ni_50.7_ SMA due to enhancement of the matrix strength by the Ti_3_Ni_4_ precipitates when the boundaries between the precipitates and matrix are coherent [18]. However, the microhardness of the Ti_49.3_Ni_50.7_ specimens decreased again after 100 h of aging because the boundaries between the precipitates and matrix became semi-coherent or incoherent when the Ti_3_Ni_4_ precipitates grew too large, leading to the deterioration of the strengthening effect.

**Figure 3:**
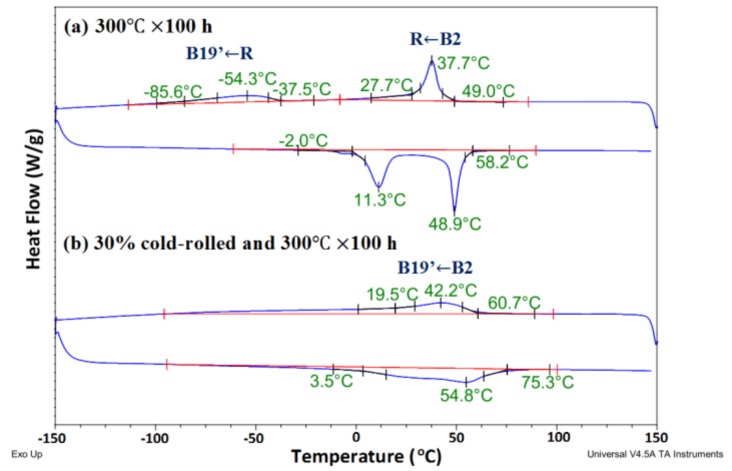
DSC result of (**a**) 300 °C × 100 h aged Ti_49.3_Ni_50.7_ SMA; (**b**) 30% cold-rolled and then 300 °C × 100 h aged Ti_49.3_Ni_50.7_ SMA.

In order to determine the thermomechanical effect on the shape memory and superelasticity properties of Ti_49.3_Ni_50.7_ SMA, a specimen was 30% cold-rolled and then aged at 300 °C for 100 h for the following experiments. Figure 3 shows the DSC results for the 30% cold-rolled and then 300 °C × 100 h aged specimen; the DSC curves of the 300 °C × 100 h aged specimen shown in Figure 1a are also plotted for comparison. As shown in Figure 3, the 30% cold-rolled and then 300 °C × 100 h aged specimen exhibited only a rather broadened one-stage B2↔B19’ martensitic transformation, with transformation peaks appearing at approximately 50 °C.

### 2.2. Shape Memory Effect Measurements

**Figure 4:**
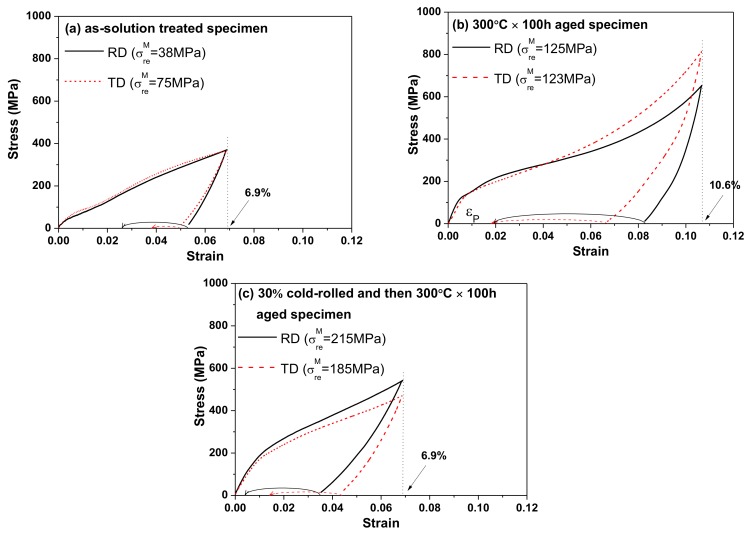
Tensile test results for (**a**) solution-treated Ti_49.3_Ni_50.7_ SMA; (**b**) 300 °C × 100 h aged Ti_49.3_Ni_50.7_ SMA; and (**c**) 30% cold-rolled and then 300 °C × 100 h aged Ti_49.3_Ni_50.7_ SMA. Each specimen was determined at temperature below M_f_ (−80 °C) along RD and TD. The curved lines in the bottom of the figures represent the recoverable strain of specimens after being heated to the temperature above Af (100 °C). RD: rolling direction; TD: transverse direction.

Figure 4a,c shows the tensile test results for the solution-treated, 300 °C × 100 h aged, and 30% cold-rolled and then 300 °C × 100 h aged rolling direction (RD) and transverse direction (TD) specimens, respectively. All specimens were tensile tested at −80 °C (below Mf temperature), and the curved lines at the bottoms of the figures represent the recoverable strain (ε_re_) of specimens after being heated to 100 °C (above Af temperature). In Figure 4, σ_re_^M^ represents the stress for martensite reorientation, and ε^M^_total_ (the total recoverable strain) equals 6.9% minus ε_p_ for the solution-treated specimen and the 30% cold-rolled and then 300 °C × 100 h aged specimen, and 10.6% minus ε_p_ for the 300 °C × 100 h aged specimen. Here, ε_p_ is the permanent (unrecoverable) strain after the specimen was heated to 100 °C, presented as an example in Figure 4b. The ε^M^_total_ values of the specimens determined from Figure 4 are listed in Table 1. In Table 1, the theoretical recoverable strain values, which were obtained from the theoretical calculations depicted in the following discussion section, are also listed here.

As shown in Figure 4, both the 300 °C × 100 h aged specimen and the 30% cold-rolled and then 300 °C × 100 h aged specimen possessed high σ_re_^M^ values of 125 MPa (RD)/123 MPa (TD) and 215 MPa (RD)/185 MPa (TD), respectively. On the other hand, the solution-treated specimen had a low σ_re_^M^ value of 38 MPa (RD)/75 MPa (TD). This indicates that the strength of Ti_49.3_Ni_50.7_ SMA with or without cold-rolling was significantly improved by the 300 °C × 100 h aging treatment. This phenomenon can be explained by the fact that low-temperature aging (<600 K) typically increases the density of Ti_3_Ni_4_ precipitates in Ni-rich TiNi SMAs, which provides more pinning points to hinder dislocation movement [5]. In addition, it has been demonstrated that annealing cold-rolled TiNi SMA at 200 °C to 600 °C is sufficient to nullify the martensite stabilization, but the dislocations induced by cold-rolling still remain inside the alloys [3]. These dislocations not only raise the required critical stress for slip, but also serve as pinning points to the moving twin boundaries. Therefore, the thermomechanical treatment of 30% cold-rolling and then 300 °C × 100 h aging was the most beneficial method to strengthen Ti_49.3_Ni_50.7_ SMA in this study. However, as shown in Table 1, despite the fact that the 30% cold-rolled and then 300 °C × 100 h aged specimen possessed the highest strength, it was the 300 °C × 100 h aged specimen that achieved the largest ε^M^_total_ value of approximately 8.8%. This unexpected result contradicts our intuitive understanding, suggesting that the strengthening effect is not the only factor that determines the shape memory ability of SMAs.

### 2.3. Superelasticity Measurements

**Figure 5:**
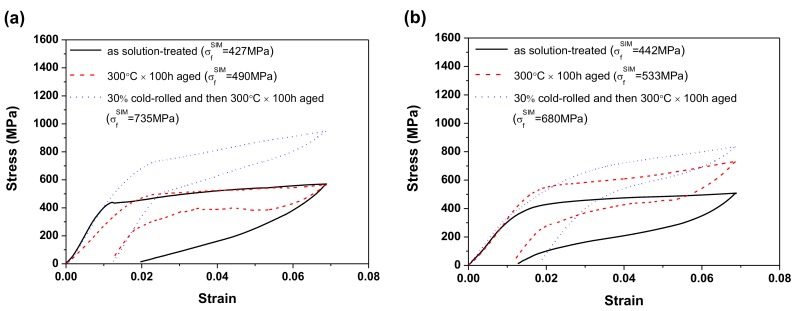
Superelasticity results for solution-treated Ti_49.3_Ni_50.7_ SMA, 300 °C × 100 h aged Ti_49.3_Ni_50.7_ SMA, and 30% cold-rolled and then 300 °C × 100 h aged Ti_49.3_Ni_50.7_ SMA determined along (**a**) RD and (**b**) TD at a temperature of 15 °C above Af.

Figure 5a,b shows the results of superelasticity measurements of RD and TD specimens, respectively, for the solution-treated, 300 °C × 100 h aged, and 30% cold-rolled and then 300 °C × 100 h aged specimens. In Figure 5, each specimen was measured at a temperature 15 °C above its Af temperature. Figure 5 shows that the 30% cold-rolled and then 300 °C × 100 h aged specimen had a higher stress value (approximately 710 MPa) to induce the stress-induced martensite (SIM) (σ_f_^SIM^) than did the solution-treated specimen (σ_f_^SIM^ was approximately 510 MPa) and 300 °C × 100 h aged specimen (σ_f_^SIM^ was approximately 435 MPa). This feature demonstrates that the 30% cold-rolled and then 300 °C × 100 h aged Ti_49.3_Ni_50.7_ SMA had the highest strength of all the specimens in terms of superelasticity measurement. Figure 5 also shows that the transformation stress of the 30% cold-rolled and then 300 °C × 100 h aged specimen increased as the transformation strain increased in the SIM region. This increase occurred because the Ti_3_Ni_4_ precipitates and dislocations that formed during the cold-rolling and aging treatment caused inhomogeneous martensitic transformation during the loading/unloading processes. The dual-phase structure in the SIM region also caused the hardening of the alloy because of the interaction of martensite correspondence variants and the blockage of existing martensite boundaries [16,17].

### 2.4. Orientation Distribution Functions (ODF) Calculations

**Figure 6:**
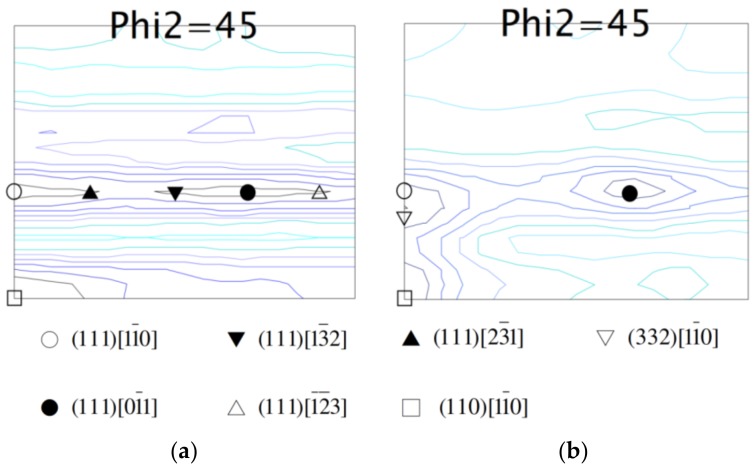
*φ*_2_ = 45° section of orientation distribution functions (ODF) for (**a**) solution-treated Ti_49.3_Ni_50.7_ SMA and (**b**) 30% cold-rolled Ti_49.3_Ni_50.7_ SMA.

Figure 6a,b shows the ODF *φ*_2_ = 45° results of the solution-treated specimen and the 30% cold-rolled specimen, respectively. Only the results of *φ*_2_ = 45° are presented in Figure 6 because most of the preferred orientations are conveniently observed in this section [8]. According to Figure 6a, the solution-treated specimen exhibited a $(110){\left[1\bar{1}0\right]}_{B2}$ texture and a {111}<uvw>_B2_ texture spreading from the line of *φ* = 55°. The ODF result of the 300 °C × 100 h aged specimen is not presented here because both B2 phase and R-phase coexisted in this alloy at room temperature. Fortunately, according to previous studies [8,20,21], it has been demonstrated that the types of textures generated by hot/cold-rolling are hardly affected by the subsequent heat-treatment. Therefore, it is reasonable to assume that the textures of the 300 °C × 100 h aged specimen should be similar to those of the solution-treated one. Figure 6b shows that the 30% cold-rolled Ti_49.3_Ni_50.7_ SMA had the major textures of $(111){\left[1\bar{1}0\right]}_{B2}$, $(111){\left[0\bar{1}1\right]}_{B2}$, $(110){\left[1\bar{1}0\right]}_{B2}$, and $(332){\left[1\bar{1}0\right]}_{B2}$ with similar intensities. Again, it is reasonable to suggest that the 30% cold-rolled and then 300 °C × 100 h aged specimen should have the same textures. Table 1 lists the determined textures of the solution-treated, 300 °C × 100 h aged, and 30% cold-rolled and then 300 °C × 100 h aged specimens for comparison.

## 3. Discussion

In order to elucidate the effects of thermomechanical treatment and its derivative textures on the shape recovery ability of the SMAs, the theoretical recoverable strains of Ti_49.3_Ni_50.7_ SMA were calculated in this study. Previous studies reported that the transformation strain calculated from the lattice deformation matrix could be applied as the maximum recoverable strain [6,7,9,16]. This method has been modified to calculate the recoverable strain for polycrystals and thin films with given textures [7,22,23,24,25]. According to the lattice deformation theory, *E^(i)^* is defined as the transformation strain matrix of a material when it transforms from austenite to the *i*th variant of martensite and is assumed to be recoverable. For a polycrystal, the orientation of the grain at point *x* is given by a rotation *R(x)* relative to a reference coordinate. The transformation strain matrix associated with the *i*th variant of martensite in the grain *x* is $E^{\left(i\right)}(x)=R(x)E^{\left(i\right)}R^{T}(x)$. Here, *R^T^* is the transpose of *R*. Consider a polycrystal in its self-accommodated martensite state, subjected to a uniaxial tensile load σ in the direction $\hat{e}$; the real recoverable strain $\varepsilon_{R}=\max\hat{e}\cdot E\hat{e}$. However, $\varepsilon_{R}$ cannot be directly calculated because the *E* of a specimen is unknown. Therefore, an *inner bound* ${\varepsilon_{R}}^{i}$ and an *outer bound* ${\varepsilon_{R}}^{o}$ are applied to estimate this real recoverable strain. In the calculation of the *inner bound* ${\varepsilon_{R}}^{i}$, each pair of martensite variants is supposed to be twin-related, while in the calculation of the *outer bound* ${\varepsilon_{R}}^{o}$, the twin-relations among martensite variants are not considered. The relation among *inner bound* ${\varepsilon_{R}}^{i}$, *outer bound* ${\varepsilon_{R}}^{o}$, and real recoverable strain $\varepsilon_{R}$ is $\varepsilon_{R}^{i}\leq \varepsilon_{R}\leq \varepsilon_{R}^{o}$. In the present study, the *outer bound* ${\varepsilon_{R}}^{o}$ of specimens is calculated, since it provides a good value relation among textures [7]. Thus, the transformation strain matrix for a polycrystal is:

(1) $$E=\sum_{i=1}^{g}\mu_{i}E_{i}=\sum_{i=1}^{g}\mu_{i}R_{i}(\sum_{j=1}^{N}\lambda_{j}^{i}E^{\left(j\right)})R_{i}^{T}$$

where *μ_i_* is the volume fraction of grain *i* and *λ^i^_j_* is the portion of material in grain *i* with the transformation strain matrix *E*^(*j*)^. Accordingly, the maximum theoretical recoverable strain of the specimen extended along direction $\hat{e}$ can be calculated from the lattice deformation matrix by Equation (2):

(2) $$\varepsilon_{R}^{o}=\max<\hat{e},E\hat{e}>=\max<\hat{e},\sum_{i=1}^{g}\mu_{i}R_{i}(\sum_{j=1}^{N}\lambda_{j}^{i}E^{\left(j\right)})R_{i}^{T}\hat{e}>,\;\lambda_{j}^{i}\geq 0,\;\sum_{j=1}^{N}\lambda_{j}^{i}=1\;=\sum_{i=1}^{g}\mu_{i}\{\max_{j=1\sim N}<{\hat{v}}_{i},E^{\left(j\right)}{\hat{v}}_{i}>\},\;{\hat{v}}_{i}=R_{i}^{T}\hat{e}$$

where ${\hat{v}}_{i}$ is the tensile direction for grain *i*.

*E*^(*1*)^ for Ti_49.4_Ni_50.6_ SMA from B2 to B19’ martensite [26,27] is:

$$\left[\begin{array}{ccc} \alpha & \delta & \varepsilon \\ \delta & \alpha & \varepsilon \\ \varepsilon & \varepsilon & \beta \end{array}\right]$$

in which α = 0.0243, β = −0.0437, δ = 0.0580, and ε = 0.0427. This matrix is applied to our calculations as the transformation strain matrix for Ti_49.3_Ni_50.7_ SMA. In the case of TiNi SMAs, the number of martensite lattice correspondence variants (*N*) is 12; therefore, *E*^(*2*)^–*E*^(*12*)^ can be calculated from *E*^(*1)*^ by these relations indicated in Reference [28]. 

From Equation (2) and the determined textures listed in Table 1, the theoretical recoverable strains of both the solution-treated Ti_49.3_Ni_50.7_ specimen and the 300 °C × 100 h aged specimen are calculated as 9.10% along RD and 8.09% along TD, since they possess identical textures. In the same manner, the theoretical recoverable strains of the 30% cold-rolled and then 300 °C × 100 h aged specimen can be calculated as 8.23% along RD and 7.34% along TD. The calculated theoretical recoverable strains for each specimen are summarized in Table 1. According to Table 1, the theoretical recoverable strains of the solution-treated specimen and the 300 °C × 100 h aged one are higher than those of the 30% cold-rolled and then 300 °C × 100 h aged specimen, indicating that the textures of the solution-treated specimen and the 300 °C × 100 h aged one are more favorable to the shape recovery ability of this SMA.

However, as shown in Table 1, the experimentally determined recoverable strains (ε^M^_total_) of the solution-treated specimen were much lower than expected. This feature could be attributed to the fact that the solution-treated specimen was the least strengthened of the three, indicating that slip deformation was easier in the solution-treated specimen. Table 1 also shows that the calculated theoretical recoverable strains of the 300 °C × 100 h aged specimen were identical to those of the solution-treated one; however, the experimental ε^M^_total_ values of the 300 °C × 100 h aged specimen were much higher than those of the solution-treated one. They were higher because the formation of Ti_3_Ni_4_ precipitates in the 300 °C × 100 h aged specimen hindered the slip deformation during the tensile test. Furthermore, the coherent stress fields around Ti_3_Ni_4_ precipitates could also facilitate the shape recovery of the alloy.

According to Table 1, the experimental ε^M^_total_ values of the 30% cold-rolled and then 300 °C × 100 h aged specimen were also lower than those of the 300 °C × 100 h aged one, even though its σ_re_^M^ and σ_f_^SIM^ values were higher than those of the other two specimens. They were higher because the high strengthening effect exhibited in the 30% cold-rolled and then 300 °C × 100 h aged specimen not only obstructed the movement of dislocations, but also hindered the movement of martensite boundaries. Moreover, as demonstrated in Figure 5, the slope of the stress-strain curve for the 30% cold-rolled and then 300 °C × 100 h aged specimen exhibits an obvious change before reaching the SIM region. This change indicates that the movements of martensite boundaries may be obstructed, causing plastic deformation before the formation of SIM to compensate for the external deformation.

## 4. Experimental Procedures

The Ti_49.3_Ni_50.7_ SMA used in this study was prepared from raw materials of titanium and nickel (both with 99.99 wt % purity) with six cycles of remelting in a vacuum arc remelter (Series 5 Bell Jar, Centorr Vacuum Ind., Nashua, NH, USA), with pure titanium used as a getter in ultrahigh purity argon gas. The weight loss during the remelting was less than 1 × 10^−5^. The as-melted ingot was hot-rolled at 900 °C into a plate with a thickness of about 1.5 mm and then solution-treated at 900 °C for 1 h. The oxidation layer of the plates was chemically etched by a solution composed of HF:HNO_3_:H_2_O = 1:5:20 (in volume ratio), and then polished with sandpaper. The solution-treated Ti_49.3_Ni_50.7_ specimen was cut with a diamond saw into small specimens with dimensions of 20 mm × 15 mm × 1.2 mm. The specimens were sealed into evacuated quartz tubes and aged at 300 °C in a furnace (BLUE M 894, Lindberg/MPH, Riverside, MI, USA) for different time periods before being quenched in water. The martensitic transformation behavior and transformation temperatures of the specimens were determined by differential scanning calorimetry (DSC) with TA Q10 equipment (Q-10, TA Instruments, New Castle, DE, USA) at a constant temperature rate of 10 °C/min.

The microhardness of the specimens was determined using an Akashi MVK-E Vickers tester (Model:HM-112, Mitutoyo Corp., Kanagawa, Japan) with a load of 4.9 N applied for 15 s. Seven tests were done on each specimen, and the distances between any two tests were at least five times the size of the indenter to avoid the effect of the stress field generated by the indenter’s point. The average microhardness value of each specimen was calculated from seven tests, with the largest and the smallest values excluded. Specimens for tensile tests were cut in the RD and TD before being ground into dog-bone shapes with a gauge size of 10 mm × 3.5 mm × 1.2 mm. The tensile tests were determined using a SHIMAZU AG-IS 50kN tensile test machine (AG-IS 50KN, Shimadzu Corp., Kyoto, Japan) equipped with a thermostatic chamber. During tensile tests, the strain rate was set at 1.3 × 10^−3^/s, and the specimens were loaded to strains of 6.9 or 10.6% before being unloaded to 0.5 kN. For superelasticity measurements, each specimen was tensile tested at a temperature of 15 °C above its Af temperature. For shape memory effect determinations, each specimen was tensile tested at −80 °C and then heated to 100 °C. The orientation distribution functions (ODF) of the specimens were calculated from the (200)_B2_, (110)_B2_, and (211)_B2_ pole figures, which were measured using a Rigaku TTR-AX3 X-ray diffractometer (TTR-AX3, Rigaku Corp., Tokyo, Japan). The ODF, including odd terms and ghost correction, was calculated up to an order of l_max_ = 22 by the series expansion method.

## 5. Conclusions

In this study, for polycrystal Ti_49.3_Ni_50.7_ SMA with various thermomechanical treatments, the transformation temperatures, microhardness, shape memory effect, superelasticity, and orientation distribution functions (ODF) of specimens were measured and their theoretical recoverable strains were calculated by the lattice deformation theory. Experimental results and theoretical calculations demonstrated that thermomechanically treated specimens had varied superelasticity and recoverable strains corresponding to the alloys’ different microstructures, textures, and strengths. The 300 °C × 100 h aged specimen had the highest ε^M^_total_ value because it had the most favorable textures and beneficial internal defects, such as Ti_3_Ni_4_ precipitates that formed and induced coherent stress. The 30% cold-rolled and then 300 °C × 100 h aged specimen had the highest strength and superelasticity of all the samples; however, its shape recovery ability was not as good as expected because the high strength of the alloy inhibited the movements of dislocations and martensite boundaries. This study reveals that the strength of Ni-rich TiNi SMAs can be significantly improved with appropriate thermomechanical treatments; however, such treatments also have side effects on the shape memory characteristics of the SMAs. The combination of hardness measurements and SMAs tensile response seems to show that the suppression of slip by Ti_3_Ni_4_ precipitates is critical and perhaps more important than texture.

## Figures and Tables

**Table 1:** The determined textures, ε^M^_total_ values determined from Figure 4 and theoretical calculated recoverable strains obtained from theoretical calculations depicted in discussion section of the solution-treated Ti_49.3_Ni_50.7_ SMA, 300 °C × 100 h aged Ti_49.3_Ni_50.7_ SMA, and 30% cold-rolled and then 300 °C × 100 h aged Ti_49.3_Ni_50.7_ SMA.

Specimen	Texture	RD	TD	Theoretical Recoverable Strain		ε^M^_total_	
				RD	TD	RD	TD
Solution-treated Ti_49.3_Ni_50.7_ SMA	$(111){\left[1\bar{1}0\right]}_{B2}$	${\left[1\bar{1}0\right]}_{B2}$	${\left[11\bar{2}\right]}_{B2}$	9.10%	8.09%	5.4%	3.1%
	$(111){\left[0\bar{1}1\right]}_{B2}$	${\left[0\bar{1}1\right]}_{B2}$	${\left[2\bar{1}\bar{1}\right]}_{B2}$				
	$(110){\left[1\bar{1}0\right]}_{B2}$	${\left[1\bar{1}0\right]}_{B2}$	${\left[00\bar{1}\right]}_{B2}$				
300 °C × 100 h aged Ti_49.3_N_i50.7_ SMA	$(111){\left[2\bar{3}1\right]}_{B2}$	${\left[2\bar{3}1\right]}_{B2}$	${\left[41\bar{5}\right]}_{B2}$	9.10%	8.09%	8.7%	8.8%
	$(111){\left[1\bar{3}2\right]}_{B2}$	${\left[1\bar{3}2\right]}_{B2}$	${\left[5\bar{1}\bar{4}\right]}_{B2}$				
	$(111){\left[\bar{1}\bar{2}3\right]}_{B2}$	${\left[\bar{1}\bar{2}3\right]}_{B2}$	${\left[5\bar{4}\bar{1}\right]}_{B2}$				
30% cold-rolled and then 300 °C × 100 h aged Ti_49.3_Ni_50.7_ SMA	$(111){\left[1\bar{1}0\right]}_{B2}$	${\left[1\bar{1}0\right]}_{B2}$	${\left[11\bar{2}\right]}_{B2}$	8.23%	7.34%	6.5%	5.5%
	(111)[01¯1]B2	${\left[0\bar{1}1\right]}_{B2}$	${\left[2\bar{1}\bar{1}\right]}_{B2}$				
	$(110){\left[1\bar{1}0\right]}_{B2}$	${\left[1\bar{1}0\right]}_{B2}$	${\left[00\bar{1}\right]}_{B2}$				
	$(332){\left[1\bar{1}0\right]}_{B2}$	${\left[1\bar{1}0\right]}_{B2}$	${\left[11\bar{3}\right]}_{B2}$				
